# Tolerogenic Dendritic Cells Generated with Tofacitinib Ameliorate Experimental Autoimmune Encephalomyelitis through Modulation of Th17/Treg Balance

**DOI:** 10.1155/2016/5021537

**Published:** 2016-12-13

**Authors:** Yan Zhou, Xiao Leng, Shasha Luo, Zhiwei Su, Xingyan Luo, Huijie Guo, Chunfen Mo, Qiang Zou, Yang Liu, Yantang Wang

**Affiliations:** ^1^Department of Pediatrics and Department of Emergency, West China Second University Hospital, Sichuan University, Chengdu 610041, China; ^2^Department of Immunology, School of Basic Medical Sciences, Chengdu Medical College, Chengdu 610083, China

## Abstract

It is well known that dendritic cells (DCs) play a pivotal role in triggering self-specific responses. Conversely, tolerogenic DCs (tolDCs), a specialized subset, induce tolerance and negatively regulate autoreactive responses. Tofacitinib, a Janus kinase inhibitor developed by Pfizer for treatment of rheumatoid arthritis, is probable to be a promising candidate for inducing tolDCs. The aims of this study were to evaluate the effectiveness of tolDCs induced by tofacitinib in a myelin oligodendrocyte glycoprotein- (MOG-) specific experimental autoimmune encephalomyelitis (EAE) model and to investigate their effects on Th17/Treg balance in the animal model of multiple sclerosis (MS). Our results revealed that tofacitinib-treated DCs maintained a steady semimature phenotype with a low level of proinflammatory cytokines and costimulatory molecules. DCs treated by tofacitinib also induced antigen-specific T cells hyporesponsiveness in a concentration-dependent manner. Upon intravenous injection into EAE mice, MOG pulsed tolDCs significantly dampened disease activity, and adoptive cell therapy (ACT) disturbed Th17/Treg balance with a remarkable decrease of Th1/Th17 cells and an increase in regulatory T cells (Tregs). Overall, DCs modified by tofacitinib exhibited a typical tolerogenic phenotype, and the antigen-specific tolDCs may represent a new avenue of research for the development of future clinical treatments for MS.

## 1. Introduction

Dendritic cells (DCs) are the most potent antigen-presenting cells (APC) for naive T cells that bridge the innate and adaptive immunity in autoimmune diseases [1]. Mature DCs (mDCs) provide self-antigen-MHC complexes (signal 1) and costimulatory molecules (signal 2) for activation of antigen-specific T cells. In addition, mDCs also provide proinflammatory cytokines (signal 3) to shape the immune response by priming the differentiation of naïve CD4+ T cells into different T helper cells [2, 3]. Tolerogenic DCs (tolDCs) which show a typical tolerogenic phenotype with normal “signal 1” but weak “signal 2” and aberrant “signal 3” has the potential to induce tolerance. The recent studies suggested that “signal 1” alone leads to inactivation of the autoreactive T cells by anergy or deletion, and aberrant “signal 3” controls CD4+ T cell fate toward a regulatory phenotype [1, 4]. Immune tolerance restoration by adoptive transfer of tolDCs has been a promising therapeutic strategy for autoimmune diseases [5, 6]. The therapeutic effect of dexamethasone/vitamin D3-modified tolDCs has been confirmed in established collagen-induced arthritis mice for reduced disease activity [7]. TolDCs generated from relapsing-remitting multiple sclerosis (MS) patients using vitamin D3 can also induce stable and antigen-specific hyporesponsiveness in autoreactive T cells [8].

Pharmacological immunosuppressive agents have proved to be valuable tools for inducing tolDCs, and the category of immune-inhibitory molecules is expected to enlarge as more compounds could be evaluated for the ability to modulate the maturation of DCs [9, 10]. Tofacitinib is a selective inhibitor of Jak1 and Jak3 which has been approved for the treatment of moderate to severe rheumatoid arthritis [11, 12]. Recent studies have found that Jak1 is involved in induction of costimulators on the surface of DCs, and abatacept as a Jak1/Jak2 inhibitor was also able to suppress CD80/86 expression [13, 14]. Bone marrow-derived dendritic cells (BMDCs) obtained from Jak3-null mice also showed reduced expression of costimulatory molecules and impaired maturation [15]. In addition to directly suppressing the production of cytokines and proliferation of T cells, tofacitinib decreased T cell stimulatory capability of human monocyte-derived DCs through Jak1/Jak3 [16, 17].

Therapies for MS that inhibit the immunogenic characters of DCs through the blockade of “signal 2” and “signal 3” are currently being pursued [18, 19]. In this work, we showed that tofacitinib prevented activation of the immune system through the modulation of the function of murine BMDCs. Tofacitinib modified BMDCs (Tofa-DCs) expressed low levels of costimulatory molecules and proinflammatory cytokines. Furthermore, through adoptive transfer of myelin oligodendrocyte glycoprotein (MOG)_35-55_ loaded Tofa-DCs to the mice with established experimental autoimmune encephalomyelitis (EAE), Th17 cells from splenocytes of treated mice decreased significantly and Tregs increased by contrast, while a reduction in disease severity and progression was observed. The cell therapy of antigen-specific tolDCs may represent a new avenue of research for the development of future clinical treatments that do not disturb the normal immune system.

## 2. Materials and Methods

### 2.1. Mice

Wild-type (WT) C57BL/6 (6–12 weeks of age) mice were purchased from Beijing Vital River Laboratory Animal Technology Co., Ltd. All mice were bred and maintained in a specific pathogen-free environment at the experimental animal center of Chengdu Medical College. All the experimental protocols were approved by the guidelines of the Animal Ethics Committee of Chengdu Medical College. C57BL/6 mice with EAE were used as animal models of human MS.

### 2.2. Generation of Murine Bone Marrow-Derived Dendritic Cells (BMDCs)

BMDCs from female C57BL/6 mice (6–8 weeks of age) were generated from BM progenitors, as previously described [20]. Briefly, both ends of the femur and tibia bones were cut with scissors, and the marrow was flushed out and passed through a nylon mesh to remove small pieces of bone and debris. On day 1, 1 × 10^6^ cells were counted and plated in 100-mm Petri dishes containing complete RPMI 1640 with granulocyte-macrophage colony-stimulating factor (GM-CSF) (10 ng/mL) and IL-4 (10 ng/mL) (PeproTech, Rocky Hill, NJ, USA). Half-medium changes took place on days 3 and 6. For maturation, 6-day-old cultures were stimulated with 100 ng/mL LPS (Sigma-Aldrich, St. Louis, MO, USA), and, 24 hours later, they were analyzed by FACS or purified with CD11c+ beads (Miltenyi Biotec, San Diego, CA, USA). In all experiments, DCs were pretreated for 12 hours with increased concentrations of tofacitinib (Tofa, Selleck Chemical LLC, TX, USA) and dissolved in dimethylsulfoxide (DMSO) that was less than 0.025% or vehicle alone before treatment with LPS.

### 2.3. Enzyme-Linked Immunosorbent Assay (ELISA)

During DCs differentiation, the culture supernatants of Tofa-DCs were collected after the cells were stimulated with LPS for 24 hours. For evaluation of cytokine secretion, ELISA kits for IL-6, IL-12, IL-23, IL-10, IL-1*β*, and TNF-*α* (eBioscience, San Diego, CA, USA) were used according to the manufacturer's instructions. Plates were read at 450 nm (PowerWave XS, BioTek, USA).

### 2.4. Induction of EAE

Twelve-week female C57BL/6 mice were subcutaneously immunized at the dorsal flanks with 200 *μ*g MOG_35-55_ peptide (Chinese Peptide Co., Ltd., Shanghai, China) emulsified in complete Freud's adjuvant (CFA) supplemented with 4 mg/mL heat-killed* Mycobacterium tuberculosis* (Chondrex, Redmond, WA, USA) at a 1 : 1 ratio. In addition, 500 ng pertussis toxin (PTx) was injected i.p. on days 0 and 2. Clinical symptoms were monitored daily after immunization. One group of mice received adjuvant and PTX only and served as the control. The clinical score was graded as follows: 0, no clinical signs; 1, paralyzed tail; 2, loss in coordinated movement, hind limb paresis; 3, both hind limbs paralyzed; 4, forelimbs paralyzed; and 5, moribund [21].

### 2.5. Adoptive Transfer Experiments

Immature DCs treated with tofacitinib (1 *μ*M) were pulsed or unpulsed for 24 hours with 20 g/mL MOG_35-55_ and 100 ng/mL LPS, and cells (1 × 10^6^ cells/mouse) were transferred i.v. into mice on days 7, 11, and 15 after EAE induction (day 0). Vehicle mice were treated with PBS.

### 2.6. Histological Analysis

For histological staining, mice were anesthetized and perfused with PBS (pH 7.4), followed by 4% (w/v) paraformaldehyde. Spinal cord samples were then fixed in 4% (w/v) paraformaldehyde overnight. Paraffin-embedded sagittal sections of cervicothoracic spinal cord were stained with hematoxylin and eosin (H&E) and examined for cellular infiltration or Luxol Fast Blue (LFB) for determining demyelination.

### 2.7. Coculture Experiments

For coculture experiments, CD4+ T cells were isolated from MOG-immunized mice using a T cell isolation kit (Miltenyi) from a single spleen-cell suspension according to the manufacturer's instructions. To examine cell proliferation, we used the carboxyfluorescein diacetate succinimidyl ester (CFSE) division method. CD4+ T cells were labeled with CFSE (1.2 *μ*M) (Invitrogen, Carlsbad, CA, USA) for 10 minutes at 37°C, washed twice with complete RPMI 1640 medium, and finally cultured with MOG_35-55_ pulsed-Tofa-DCs at three DC : T cells ratios (1 : 10, 1 : 30, and 1 : 100). After 72 hours, the triplicate cultures were analyzed by flow cytometry.

### 2.8. Flow Cytometry and Intracellular Cytokine Staining

DCs phenotype after maturation was analyzed for their surface marker expression by flow cytometry. All staining procedures were performed in phosphate buffered saline (PBS) containing 2 mM ethylenediaminetetraacetic acid (EDTA) and 0.1% BSA, and then 2 × 10^5^ cells were incubated in the dark for 30 minutes on ice with specific conjugated antibodies for CD11c, CD83, CD40, CD80, CD86, and MHC II or with matching isotype controls. All antibodies (Abs) were purchased from BioLegend (San Diego, CA, USA).

To analyze effector CD4+ T cell populations, spleen cells from EAE mice or T cells from coculture experiments were prepared from cell cultures and were stained at 4°C in PBS containing 2% fetal bovine serum (FBS) and 1% EDTA after blocking Fc R with 2.4G2 (Mouse BD Fc Block™). For IFN-*γ* and IL-17 intracellular staining, fixation and permeabilization buffers with GolgiPlug™ (BD Pharmingen, San Jose, CA, USA) were utilized. Cells were stained with mouse anti-CD4 fluorescein isothiocyanate (FITC) antibody and were then incubated with PE-conjugated anti-IFN-*γ* and Alexa Fluor® 647-conjugated anti–IL-17. Tregs were detected using the Transcription Factor Buffer Set (BD Pharmingen) and were then incubated with PE-conjugated anti-Foxp3 and APC-conjugated anti-CD25. All Abs were purchased from BD Pharmingen. Stained cells were analyzed with flow cytometer Accuri™ C6 (BD Biosciences, San Diego, CA, USA).

### 2.9. Statistical Analysis

Statistics were calculated with the Mann-Whitney *U* test (no Gaussian distribution). The difference among all experiment groups including cell and animal results was determined by one-way analysis of variance (ANOVA) with Tukey's post hoc test (Gaussian distribution). Spearmen's rank test was utilized to test the association between clinical score and populations of T helper cells analyzed in animal model (no Gaussian distribution). Statistical analysis was performed with the help of Prism 6.0 software (GraphPad Software, San Diego, CA, USA). Data are reported as mean ± SEM. *P* values < 0.05 were considered significant.

## 3. Results

### 3.1. Tofa-DCs Retained the Tolerogenic Phenotype

**Figure 1:**
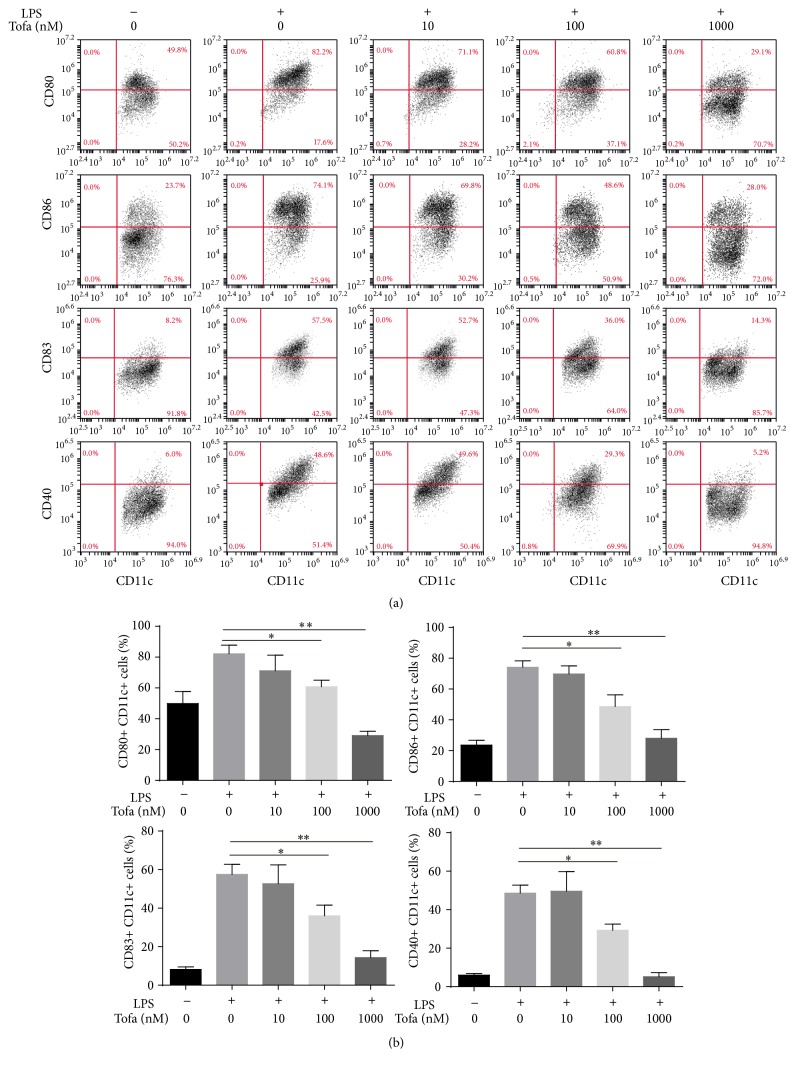
Analysis of Tofa-DCs surface phenotype by flow cytometry. (a) Tofa-pretreated DCs were cultured in the presence or in the absence of LPS (100 ng/mL) for 24 hours. Surface expressions of CD11c, CD83, CD40, CD80, and CD86 on DCs were analyzed by flow cytometry. (b) The frequencies of CD83, CD40, CD80, or CD86 positive cells were determined. The representative results of three independent experiments with similar findings are shown. Statistical testing was performed using the Mann-Whitney test. Data are means ± SEM, ^*∗*^ *P* < 0.05, and ^*∗∗*^ *P* < 0.01.

Previous studies have reported that tofacitinib decreased the expression of CD80/CD86 in LPS-stimulated human monocyte-derived DCs [16]. In this experiment, we analyzed whether tofacitinib was able to modulate the differentiation profile of murine BMDCs. DCs were generated from naïve mouse bone marrow cells and were treated with tofacitinib before LPS-induced maturation to generate tolDCs. LPS-activated DCs that were not exposed to tofacitinib were named mature DCs (mDCs), whereas the untreated DCs were defined as immature dendritic cells (imDCs) and were used as a control population. As expected, imDCs expressed significantly lower levels of CD40, CD83, CD80, and CD86 compared to mDCs, whereas the tolDCs treated with tofacitinib displayed a typical semimature phenotype of reduced CD40, CD83, CD80, and CD86 in a concentration-dependent manner (Figure 1).

### 3.2. Cytokine Production by Tofa-DCs

**Figure 2:**
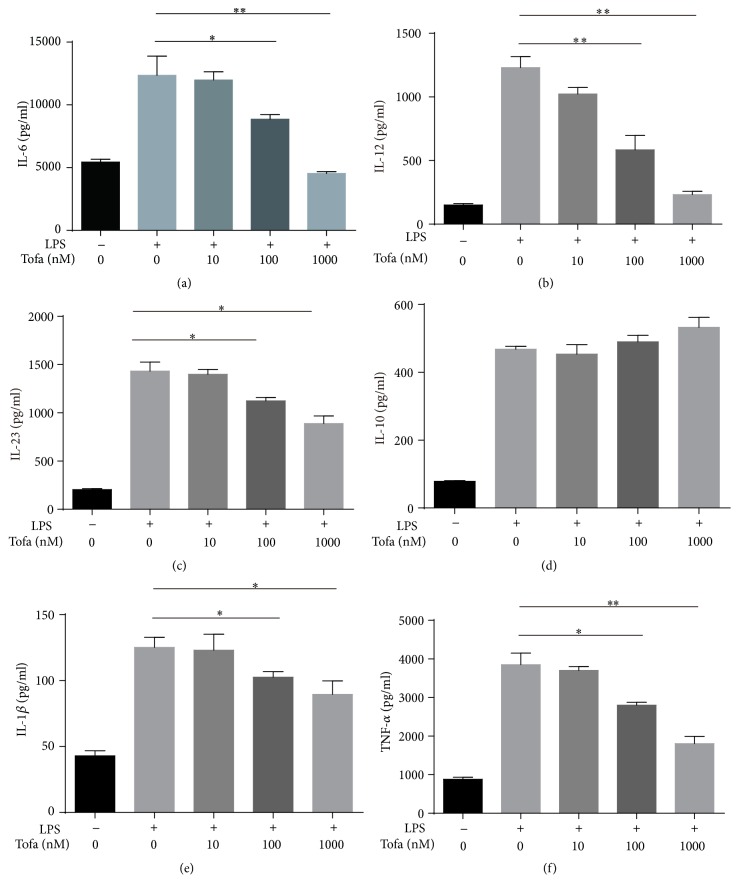
Tofacitinib inhibited secretion of proinflammatory cytokines. Levels of interleukin-6 (IL-6) (a), IL-12 (b), IL-23 (c), IL-10 (d), IL-1*β* (e), and tumor necrosis factor-*α* (TNF-*α*) (f) in supernatants containing each DCs subset were determined by enzyme-linked immunosorbent assay (ELISA). The representative results of three independent experiments with similar findings are shown. Statistical testing was performed using the Mann-Whitney test. Values represent the means ± SEM, ^*∗*^ *P* < 0.05, and ^*∗∗*^ *P* < 0.01.

The altered DC cytokine profile was also one of the mechanisms by which DCs contributed to tolerance-induced immune-network [22]. Therefore, we analyzed the Tofa-treated DCs cytokine secretion upon LPS. As expected, mDCs produced high amounts of IL-1*β*, IL-6, IL-12, TNF-*α*, and IL-23 in response to LPS. Tofacitinib (100, 1000 nM)-treated DCs produced markedly lower amounts of IL-1*β* and IL-23 compared with mDCs. Moreover, the production of IL-6, IL-12, and TNF-*α* induced by LPS was shown to be significantly lower for Tofa-treated DCs compared with mDCs. Tofacitinib did not affect the secretion of IL-10, even at a high concentration as 1000 nM (Figure 2).

### 3.3. The Adoptive Transfer of Tofa-DCs Modulated EAE

**Figure 3:**
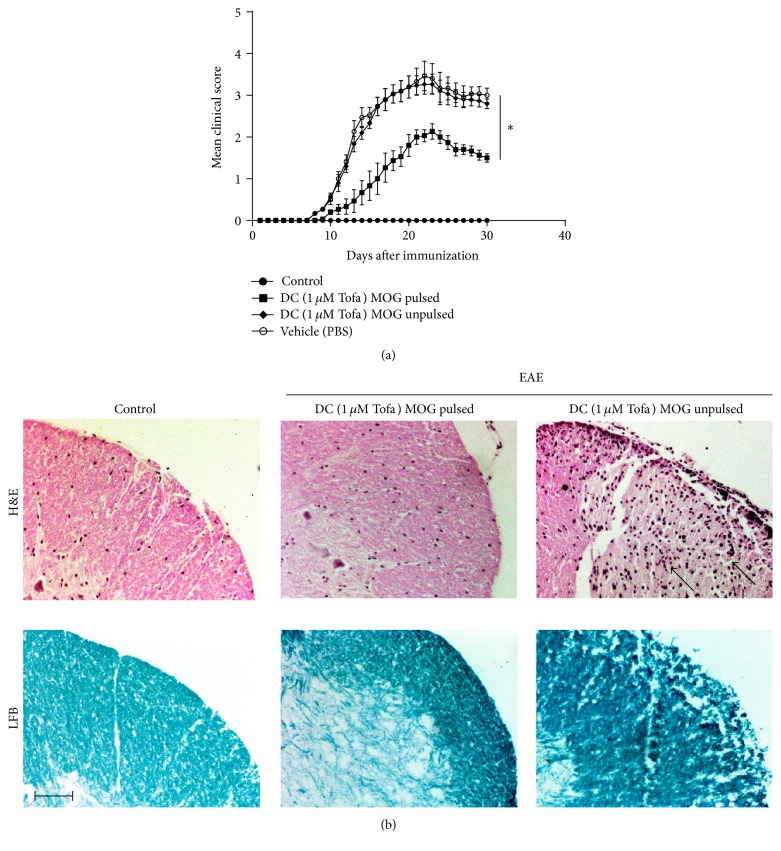
Tofa-treated DCs treatment ameliorated EAE. (a) C57BL/6 mice were immunized with MOG_35-55_ peptide emulsified in CFA containing* M. tuberculosis*. Tofa-treated DCs loaded or unloaded MOG_35-55_ were injected intravenously into mice with EAE on days 7, 11, and 15 after immunization. EAE scores were determined daily after disease onset in the four groups with various treatment conditions. The mean EAE scores ± SEM are shown. The difference was significant at the onset of disease on day 11 and persisted until the chronic phase between the PBS group (vehicle) and Tofa-treated DCs pulsed with MOG_35-55_ group. ^*∗*^ *P* < 0.05 by ANOVA/Tukey's post hoc test. In addition, Tofa-treated DCs unpulsed with MOG_35-55_ could not improve disease activity of EAE, similar to that of the PBS group. Results represent the average disease score of eight mice per group. (b) H&E staining and Luxol Fast Blue staining of the paraffin sections of the spinal cords isolated from EAE mice, which were injected with Tofa-treated DCs pulsed or unpulsed with MOG_35-55_ on day 23 after immunization. Arrows indicate inflammatory cell infiltration. Scale bar, 100 *μ*m (magnification 100x).

**Figure 4:**
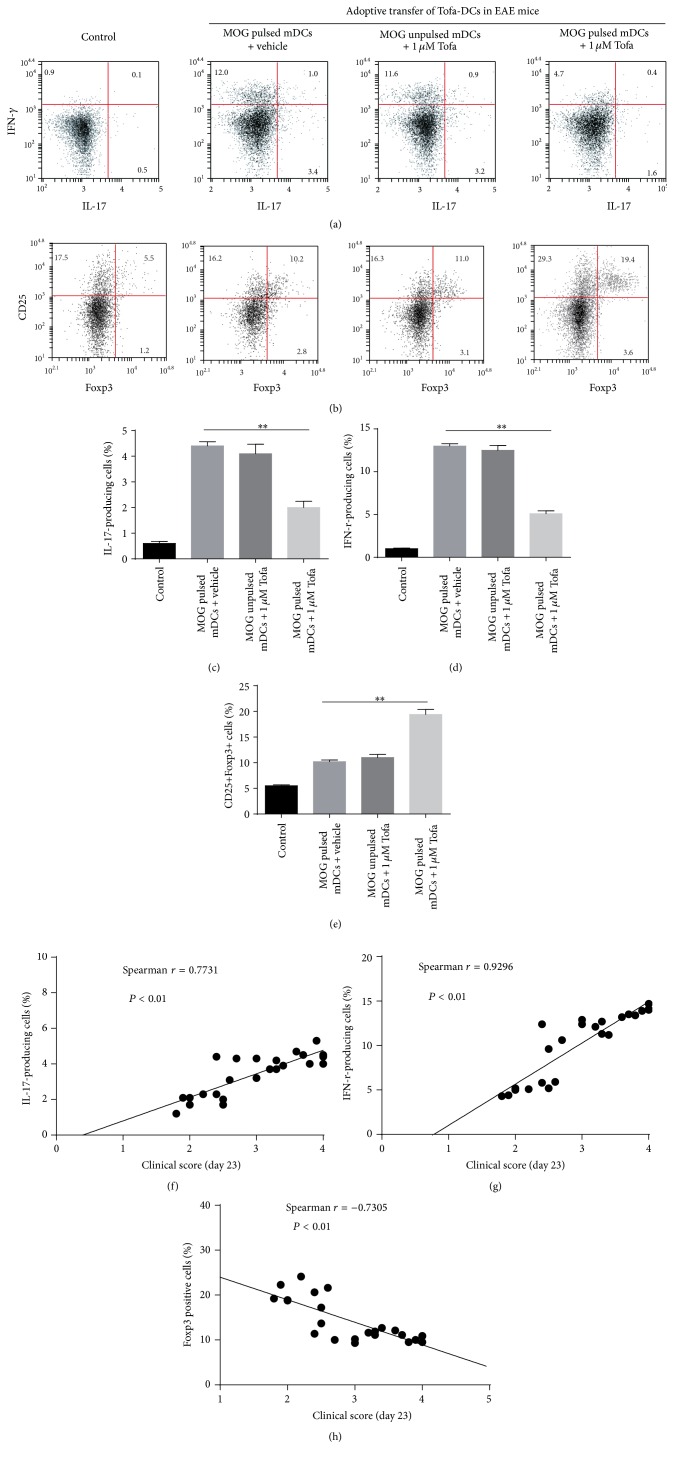
Adoptive transfer of Tofa-treated DCs decreased the proportion of IL-17+ and IFN-*γ*+ T cells and induced expansion of CD25+Foxp3+ Tregs from EAE mice. CD4+ T cells were sorted from spleens of EAE mice on day 23 after immunization, IL-17, IFN-*γ* (a), and Foxp3 (b) were measured by intracellular cytokine staining, and a set of representative data of eight mice per group was presented. Histograms showed the percentage of IL-17A+ (c), IFN-*γ*+ (d), and CD25+Foxp3+ cells (e). The values represent the means ± SEM, ^*∗*^ *P* < 0.05, and ^*∗∗*^ *P* < 0.01 by ANOVA/Tukey's post hoc test. The correlations between clinical scores (day 23) and the frequencies of IL-17-producing cells, IFN-r-producing cells and CD25+Foxp3+ cells in spleen of animal models (MOG pulsed mDCs with vehicle group, MOG unpulsed mDCs with 1 *μ*M Tofa group, and MOG pulsed mDCs with 1 *μ*M Tofa group) were presented as (f), (g), and (h), respectively, by using Spearmen's rank test.

The above experiments suggested that Tofa-DCs altered the T cells polarization compared with mDCs (matured without tofacitinib) in vitro. To further examine the immunotherapeutic potential of tolDCs induced by tofacitinib, we analyzed the effect of Tofa-DCs treatment on EAE, an animal model of MS. Female 12-week-old C57BL/6 mice were immunized s.c. with 200 *μ*g of MOG_35-55_ emulsified in CFA (4 mg/mL* Mycobacterium tuberculosis*) and injected i.p. on days 0 and 2 with 500 ng PTx. One week after induction, the mice were randomized. For adoptive transfer treatment, 1 × 10^6^  MOG_35-55_ pulsed or unpulsed tolDCs were transferred into EAE mice on days 7, 11, and 15 after immunization (dpi 7, 11, and 15). The vehicle group was injected intravenously with PBS. The EAE mice were checked for disease clinical score after disease onset (dpi 9). The results showed that the Tofa (1000 nM)-DCs treated group had a significantly milder disease score compared with the PBS group (Figure 3(a)). Histological analysis of spinal cord sections at peak disease (dpi 23) also showed that treatment with Tofa-DCs pulsed by MOG_35-55_ caused a dramatic reduction of leukocyte infiltration in the spinal cord. Luxol Fast Blue staining also indicated less extensive demyelination in EAE mice treated with Tofa-DCs pulsed with MOG_35-55_ than that of the unpulsed group (Figure 3(b)). On the other hand, the frequencies of IFN-*γ*- and IL-17-producing cells were significantly reduced in the spleen cells of EAE mice treated with Tofa-DCs loaded MOG_35-55_, compared with the PBS group (dpi 23) (Figure 4(a)). In contrast, an increase in the percentage of CD25+Foxp3+ Tregs is presented in Figure 4(b), and splenocytes from EAE mice treated with Tofa-DCs pulsed or unpulsed with MOG_35-55_ had 19.4 ± 2.4% and 11.0 ± 1.5% Tregs (*P* < 0.01). In addition, the clinical scores (day 23) were strongly correlated with the frequencies of IL-17-producing cells (Figure 4(f)) and IFN-r-producing cells (Figure 4(g)) and negatively correlated with the population of CD25+Foxp3+ cells (Figure 4(h)) in spleen of animal models. In summary, the adoptive transfer of tolDCs induced by tofacitinib contributed to the aberrant polarization of CD4+ T helper cells and the suppression of EAE.

### 3.4. Tofa-DCs Stimulated Less Antigen-Specific T Cells Proliferation

**Figure 5:**
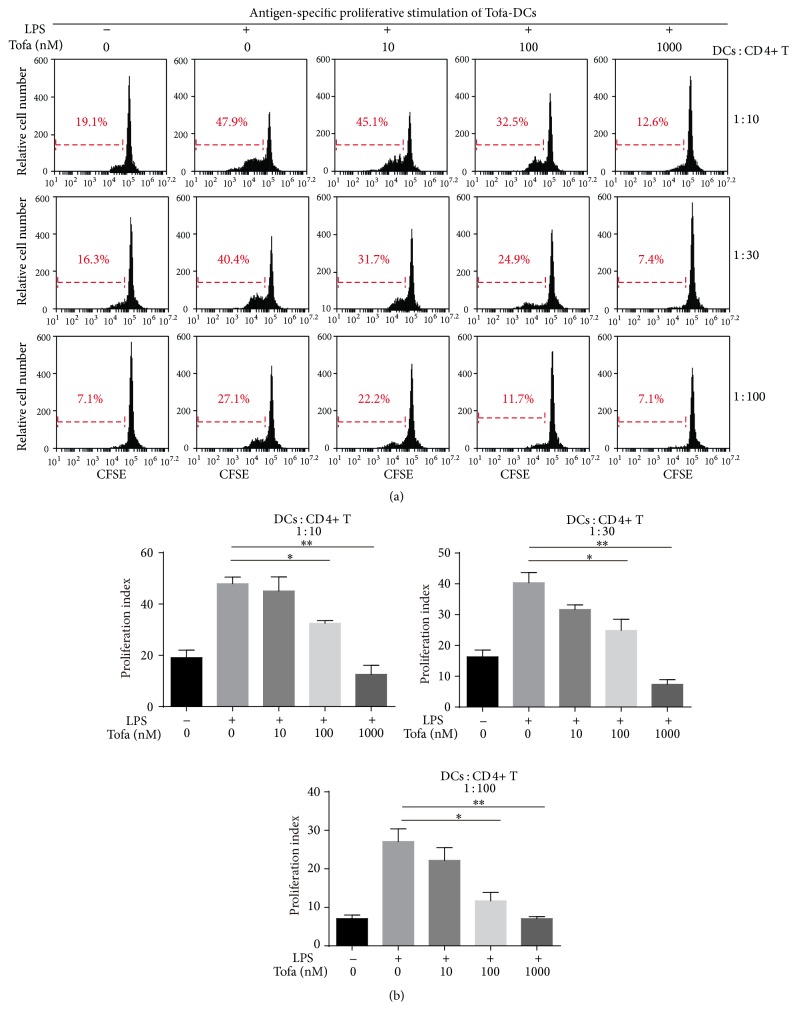
Antigen-specific T cell stimulation by MOG_35-55_ pulsed-Tofa-DCs. (a) ImDC, mDCs, and Tofa-DCs were mixed with CFSE-labeled CD4+ T cells from EAE mice for 72 hours with different ratios of DCs : CD4+ T (1 : 10, 1 : 30, and 1 : 100), and the proliferation of T cells was determined by CFSE dilution through flow cytometry. (b) Graphic shows the average ± SEM of the percentage of proliferating CFSE-T cells. The representative results of three independent experiments with similar findings are shown. Statistical testing was performed using the Mann-Whitney test. ^*∗*^ *P* < 0.05 and ^*∗∗*^ *P* < 0.01.

We next investigated the immunoregulatory capacity of Tofa-treated DCs. MOG_35-55_ pulsed-Tofa-DCs under various conditions were cocultured with CD4+ T cells from MOG-immunized mice at three DC : T cells ratios (1 : 10, 1 : 30, and 1 : 100) for 3 days. Then T cell proliferation was analyzed by flow cytometry. mDCs were superior to Tofa (100 nM and 1000 nM)-treated DCs in inducing the proliferation of antigen-specific T cells at all three DC : T cells ratios. Tofa (1000 nM)-treated DCs could not expand CD4+ T cells at high concentrations of DCs. Overall, MOG_35-55_ pulsed-Tofa-DCs were not able to establish strong interaction with antigen-specific T cells (Figure 5), probably because they expressed a low level of costimulatory molecules and produced decreased proinflammatory cytokines.

### 3.5. Tofa-DCs Affected Th1/Th17 Cell Differentiation In Vitro

**Figure 6:**
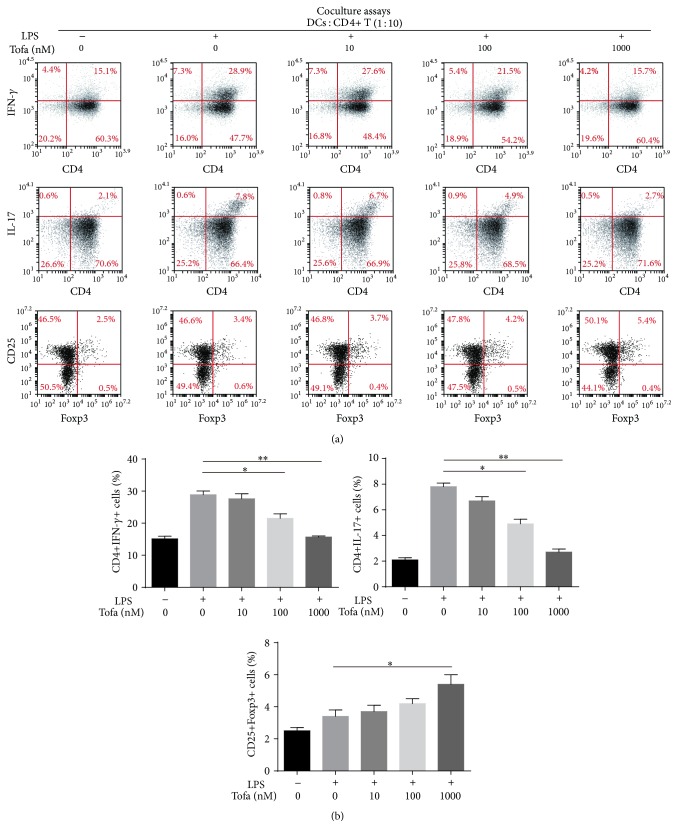
MOG_35-55_ pulsed-Tofa-DC induced the hyporesponsiveness of antigen-specific T cells. (a) Activated CD4+ T cells were harvested and the expressions of IL-17A, IFN-*γ*, and foxp3 were analyzed by intracellular staining and flow cytometry in the gate of CD4+ T cells or CD4+ CD25+ T cells. (b) Histograms showed the percentage of IL-17A, IFN-*γ* producing cells, or CD25+Foxp3+ T cells in coculture experiments under different conditions. The representative results of three independent experiments with similar findings are shown. Statistical testing was performed using the Mann-Whitney test. Values represent the means ± SEM, ^*∗*^ *P* < 0.05, and ^*∗∗*^ *P* < 0.01.

Activated CD4+ T cells were obtained from coculture experiments at a ratio of 1 : 10 (DCs : T cells), and the effects of stimulation were tested in vitro for their phenotypes and functions. As shown in Figure 6(a), culture of antigen-specific T cells with MOG_35-55_ pulsed-mDCs resulted in approximately 27% to 30% of CD4+ T cells differentiating into Th1 cells, which decreased to approximately 14% to 16% when cultured with tofacitinib (1000 nM)-treated DCs. Similarly, culture of antigen-specific T cells with MOG_35-55_ pulsed-mDCs resulted in approximately 7.1% to 8.2% of CD4+ T cells differentiating into Th17 cells, which decreased to approximately 2.5% to 3.1% when cultured with tofacitinib (1000 nM)-treated DCs. Above all, tofacitinib impaired the inducibility of mDCs for Th1/Th17 cells differentiation in a dose-dependent manner. In contrast, the frequency of CD4+CD25+Foxp3+ Tregs induced by MOG_35-55_ pulsed-Tofa-DCs at the concentration of 1000 nM was superior to that which was induced by MOG_35-55_ pulsed-mDCs (Figure 6(b)).

## 4. Discussion

MS is a chronic autoimmune disease of the central nervous system, manifesting as inflammatory demyelination and axonal loss [23]. Autoreactive T cells that recognize myelin antigen exist within the cell infiltrate and trigger a cascade of proinflammatory events resulting in the formation of the demyelinating lesion [24]. The powerful presentation of self-antigens by DCs is crucial for the priming and differentiation of self-reactive T cells [25]. Moreover, recent studies reported that DCs derived from pre-DC marrow precursor in the steady-state CNS exhibited a differentiation and antigen-presenting features similar to spleen DCs [26]. Therefore, novel therapy to target inflammatory DCs in MS would be promising.

In the current study, our in vitro data showed that tofacitinib exhibited a suppressive effect on the maturation of murine BMDCs stimulated with LPS. The tolerogenic phenotypes of tofacitinib modified BMDCs had properties similar to human monocyte-derived DCs treated with tofacitinib [16]. Tofacitinib not only prevented murine DCs increasing the expression of costimulatory molecules and activation marker such as CD80, CD86, CD40, and CD83 but also inhibited the production of proinflammatory cytokine, IL-1*β*, IL-6, IL-12, TNF-*α*, and IL-23. It was well known that costimulatory molecules were closely related to antigen presentation [27], and “signal 3” inflammatory cytokines were crucial factors for polarization of naïve CD4+ T cells. Th1 cell differentiation required higher levels of CD80 and CD86 expression and IL-12 production, and IL-23 was known to promote Th17 differentiation [28, 29].

In our coculture experiments, Tofa-DCs exhibited decreased reactivity and proliferation of antigen-specific T cells compared with mature DCs (matured without tofacitinib). MOG_35-55_ pulsed-Tofa-DCs significantly limited the expansion of Th1/Th17 cells. IFN-*γ*-producing Th1 cells have been reported to be implicated in EAE induction, and recent studies indicated that IL-17-producing Th17 cells may also play a more important role in the development of EAE. Differentiated Th17 cells secreted proinflammatory cytokines, which activated macrophages destroying myelin and damaging oligodendrocytes. Moreover, Th17 cells were capable of inducing EAE independently of other T helper cell subsets [30]. Interestingly, our data showed tofacitinib did not affect IL-10 production of LPS-stimulated DCs, but MOG_35-55_ pulsed-Tofa-DCs could polarize antigen-specific CD4+ T cells toward CD25+Foxp3+ T cells in vitro. It was possible that Tofa-DCs secreted a low level of IL-12, which could downregulate induced Tregs (iTregs) differentiation in coculture experiments [31]. Previous studies have investigated the suppressive function of iTregs both in the EAE model and in MS patients, and the adoptive transfer of Tregs to EAE mice significantly reduced disease severity [18, 32]. Given that MOG_35-55_ pulsed-Tofa-DCs functionally impaired antigen-specific T cell activation and differentiation, adoptive cell therapy (ACT) may be a potent strategy to arrest the ongoing autoimmune response in MS.

As expected, in mice with established EAE, treatment with MOG_35-55_ loaded Tofa-DCs led to a reduction in disease severity and progression through modulation of Th17/Treg balance. Histological analysis also showed that treatment with Tofa-DCs pulsed by MOG_35-55_ caused a dramatic reduction of leukocyte infiltration in the spinal cord and less extensive demyelination. Our data suggested that Tofa-DCs played a therapeutic role in ameliorating disease progression in the EAE mouse model by downregulating the Th1/Th17 population and upregulating the Treg population in vivo. Surprisingly, <100 nM tofacitinib was indicated to promote Th17 differentiation in vitro, and a low dose (15 mg/kg body weight) of tofacitinib would accelerate the onset of EAE [33]. In contrast, tofacitinib could effectively inhibit the generation of inflammatory Th17 cells stimulated by IL-1*β*, IL-6, and IL-23 [34]. Because of these contradictory results, we suggested that the adoptive transfer of tofacitinib modified DCs for the treatment of MS may be a better choice than the administration of tofacitinib directly. Moreover, Mansilla et al. reported that cryopreservation did not affect typical characters of tolerogenic dendritic cells, which made this field particularly exciting [35]. Furthermore, the major barrier of tolDC-mediated immunotherapy in autoimmune diseases was the identification of autoantigen loaded onto the tolDCs [36]. Although the search for autoantigen in patients with multiple sclerosis has been ongoing for decades and several proteins have been postulated to be potent autoantigens, such as *α* B-crystallin [37], anoctamin 2 [38], and KIR4.1 [39], the exact target autoantigen of MS remains unclear. The breakthrough in searching for specific autoantigens in patients with MS has been an urgent need for clinical and preclinical translation of tolDCs-based therapies.

## 5. Conclusions

Our data indicated that DCs modified by tofacitinib exhibited a typical tolerogenic phenotype, and Tofa-DCs pulsed with MOG_35-55_ could effectively dampen the severity and progression of EAE. This suppression of EAE was associated with remarkable decrease of Th1/Th17 cells and an increase in Tregs. The potential therapeutic effects of antigen-specific tolDCs have been confirmed in animal models of autoimmune diseases, and the encouraging results will certainly facilitate the design of future immunotherapeutic trials in patients with MS.
